# Endovascular therapy for acute ischaemic stroke: the Pragmatic Ischaemic Stroke Thrombectomy Evaluation (PISTE) randomised, controlled trial

**DOI:** 10.1136/jnnp-2016-314117

**Published:** 2016-10-18

**Authors:** Keith W Muir, Gary A Ford, Claudia-Martina Messow, Ian Ford, Alicia Murray, Andrew Clifton, Martin M Brown, Jeremy Madigan, Rob Lenthall, Fergus Robertson, Anand Dixit, Geoffrey C Cloud, Joanna Wardlaw, Janet Freeman, Philip White

**Affiliations:** 1 Institute of Neuroscience & Psychology, University of Glasgow, Queen Elizabeth University Hospital, Glasgow, UK; 2 Division of Medical Sciences, Oxford University Hospitals NHS Trust, Oxford University, Oxford, UK; 3 Robertson Centre for Biostatistics, University of Glasgow, Glasgow, UK; 4 St George's, University of London, London, UK; 5 Stroke Research Centre, UCL Institute of Neurology, University College London, London, UK; 6 Department of Neuroradiology, Queen's Medical Centre, Nottingham, UK; 7 Institute of Neuroscience, Newcastle University, Newcastle upon Tyne, UK; 8 Brain Research Imaging Centre, Centre for Clinical Brain Sciences, University of Edinburgh, Edinburgh, UK; 9 Lay member, Cornwall, UK

## Abstract

**Objective:**

The Pragmatic Ischaemic Thrombectomy Evaluation (PISTE) trial was a multicentre, randomised, controlled clinical trial comparing intravenous thrombolysis (IVT) alone with IVT and adjunctive intra-arterial mechanical thrombectomy (MT) in patients who had acute ischaemic stroke with large artery occlusive anterior circulation stroke confirmed on CT angiography (CTA).

**Design:**

Eligible patients had IVT started within 4.5 hours of stroke symptom onset. Those randomised to additional MT underwent thrombectomy using any Conformité Européene (CE)-marked device, with target interval times for IVT start to arterial puncture of <90 min. The primary outcome was the proportion of patients achieving independence defined by a modified Rankin Scale (mRS) score of 0–2 at day 90.

**Results:**

Ten UK centres enrolled 65 patients between April 2013 and April 2015. Median National Institutes of Health Stroke Scale score was 16 (IQR 13–21). Median stroke onset to IVT start was 120 min. In the intention-to-treat analysis, there was no significant difference in disability-free survival at day 90 with MT (absolute difference 11%, adjusted OR 2.12, 95% CI 0.65 to 6.94, p=0.20). Secondary analyses showed significantly greater likelihood of full neurological recovery (mRS 0–1) at day 90 (OR 7.6, 95% CI 1.6 to 37.2, p=0.010). In the per-protocol population (n=58), the primary and most secondary clinical outcomes significantly favoured MT (absolute difference in mRS 0–2 of 22% and adjusted OR 4.9, 95% CI 1.2 to 19.7, p=0.021).

**Conclusions:**

The trial did not find a significant difference between treatment groups for the primary end point. However, the effect size was consistent with published data and across primary and secondary end points. Proceeding as fast as possible to MT after CTA confirmation of large artery occlusion on a background of intravenous alteplase is safe, improves excellent clinical outcomes and, in the per-protocol population, improves disability-free survival.

**Trial registration number:**

NCT01745692; Results.

## Background

Recanalisation and reperfusion of the brain are associated with greater chance of favourable outcome after acute ischaemic stroke.^1^ Intravenous thrombolysis with recombinant tissue plasminogen activator (rtPA) improves the likelihood of recanalisation, and treatment within 4.5 hours of stroke onset is associated with significantly increased likelihood of recovery without disability by 90 days after stroke.^2^ However, among those patients with large artery occlusion (LAO) in the carotid circulation (occlusion of the intracranial internal carotid artery (ICA), or proximal middle cerebral artery (MCA)), intravenous rtPA is able to effect recanalisation in only a small proportion of patients,^3^ and despite intravenous thrombolytic therapy, a high proportion of patients with LAO stroke die or remain disabled.

Intra-arterial treatment of stroke, initially with thrombolytic drugs and then with endovascular devices designed to fragment or extract the causative thrombus, has been investigated over many years. Devices developed in the early 2000s achieved higher rates of recanalisation, but clinical outcomes were not clearly superior to those achieved by intravenous thrombolysis (IVT) alone.^4^ ^5^ The third Interventional Management of Stroke trial (IMS-3)^6^ found no difference in outcome between patients treated with IVT alone compared to IVT with additional intra-arterial treatment. The IMS-3 trial used predominantly older devices that were found subsequently to be less effective than the later stent-retriever devices^7^ ^8^ and did not have non-invasive vascular imaging to establish the presence of treatable LAO in the majority of its patients. The trial also identified a strongly time-dependent likelihood of recovery to independence, emphasising the importance of fast intervention times.^9^

A series of five trials using angiographic imaging and mechanical thrombectomy (MT) predominantly using stent-retrievers reported positive results in 2015 in favour of MT,^10–14^ with results from two further trials presented. All trials were conducted at expert stroke centres with highly efficient systems for delivery of MT and experience in this modality of treatment. Whether these findings are generalisable to countries with different healthcare systems, such as the UK, was unclear.

We undertook the PISTE trial to evaluate the efficacy of MT in addition to best medical therapy, including IVT, compared to best medical therapy alone. Recruitment to the trial was halted after review of other trial data.

## Methods

PISTE was a multicentre, randomised, controlled, parallel group trial of prospective, randomised, open, blinded end point evaluation (PROBE) design (clinicaltrials.gov NCT01745692). Ethical approvals were given by the Scotland A Research Ethics Committee (12/SS/0059) and the National Research Ethics Service Committee North East-Newcastle & North Tyneside 2 (12-NE-0315). Adult patients ≥18 years were eligible if presenting with acute supratentorial ischaemic stroke and eligible for IVT started within 4.5 hours of symptom onset. If non-invasive angiographic imaging with CT angiography (CTA) or magnetic resonance angiography showed occlusion of the intracranial ICA, M1 segment of the MCA or a single M2 MCA branch, patients were eligible for randomisation. We excluded patients with contraindications to IVT, life expectancy limited to <90 days, with chronic extracranial ICA occlusion or with extensive early hypodensity on non-contrast CT brain involving more than one-third of the MCA territory. All patients had IVT initiated at the neurovascular centre.

Neurointerventional centres were required to have a minimum of two experienced operators—with ≥10 thrombectomy procedures per centre for acute stroke treatment in the preceding 18 months, and to have extensive experience of other intracranial endovascular procedures—with centre volumes exceeding 120 per annum for the past 3 years and individual operators exceeding 120 in total; of which, at least 60 were in the preceding 18 months. Intervention was to be initiated as fast as possible after confirming eligibility, and a maximum of 90 min from start of IVT to start of the MT procedure (groin puncture) was permitted. The target vessel should have been cannulated within a maximum of 6 hours of symptom onset.

Patients were randomised 1:1 to receive best medical therapy with IVT alone, or undergo additional (adjunctive) MT with any operator-selected CE-marked device approved for intracranial clot removal. Allocation used a minimisation algorithm, including age group, stroke severity on the National Institutes of Health Stroke Scale (NIHSS) and symptom onset-to-treatment time. Randomisation was conducted using an interactive voice-response system managed by the Robertson Centre for Biostatistics, University of Glasgow.

The primary outcome was defined as the proportion of patients achieving independence at day 90 after stroke onset, based on a score of 0, 1 or 2 on the modified Rankin Scale (mRS).^15^ ^16^ Day 90 outcomes were assessed by site staff blind to treatment allocation. We defined secondary outcome measures as excellent recovery (mRS score 0–1); change in the distribution of scores on the mRS; early major neurological improvement (improvement by ≥8 points on the NIHSS or NIHSS of 0 or 1 at 24 hours after stroke); ‘home time’ (time spent in usual residence between stroke onset and day 90);^17^ the proportion of patients with recanalisation on the IST-3 CTA scale^18^ at 24 hours; mortality; and the incidence of symptomatic intracerebral haemorrhage (SICH) defined using SITS-MOST criteria^19^ as a parenchymal haematoma type 2^20^ on CT or MRI brain at 24–36 hours and a clinical worsening of ≥4 points on the NIHSS.

All imaging studies were uploaded to a central imaging repository, anonymised, validated and loaded into a web-based viewing system (Systematic Image Review System-2, SIRS-2^21^) for reading by three neuroradiologists blind to treatment allocation as well as all clinical data as a ‘core lab’ interpretation. Extent of early ischaemic change on brain imaging was defined by the Alberta Stroke Programme Early CT score (ASPECTS).^22^ The site of vessel occlusion at baseline was defined on CT angiography (CTA). Collateral circulation was graded as poor, moderate or good.^11^ The extent of thrombus was graded using the clot burden scale.^23^ Recanalisation at the end of the procedure among those allocated additional MT was graded by the modified Thrombolysis in Cerebral Infarction (mTICI) scale with reperfusion success defined as mTICI score 2b or 3. Recanalisation at 24 hours was assessed on repeat CTA using the third International Stroke Trial (IST-3) CTA score.^18^

The intention-to-treat (ITT) population consists of all patients randomised in the trial, and the per-protocol population consists of all patients in the ITT population who did not have any major protocol violation identified prior to database lock.

The primary efficacy analysis is the comparison of the primary outcome mRS ≤2 at day 90 between treatment groups using logistic regression adjusting for the minimisation factors used in the randomisation. These were age group (≤80 or >80), NIHSS score (6–12, 13–19, 20–42), time to rtPA (<3 hours, ≥3 hours) and study site. Analyses were performed identically for ITT and per-protocol populations. For the analysis, sites recruiting fewer than 10 patients were grouped together. Binary secondary outcomes were analysed analogously. mRS distribution was analysed using proportional odds logistic regression instead of logistic regression, additionally adjusting for prestroke (baseline) mRS. The number of days in usual residence between day 0 and day 90 was analysed using exact permutation tests.

The statistical analysis plan was agreed prior to database lock and unblinding. Statistical analyses have been carried out using R V.3.0.1 (R Foundation for Statistical Computing, Vienna, Austria). The significance level for the primary analysis is 0.05.

The original sample size calculation assumed that 44% of intravenous-treated and 57% of MT-treated patients would achieve mRS 0–2, based on the CTA subgroup of IMS-3.^24^ This yielded a sample size of ∼200 participants per group for 80% power, p=0.05. Since a more conservative 10% absolute increase in independent recovery would have been clinically worthwhile, a sample size of 400 participants per group was originally planned (assuming 45% and 55% mRS 0–2 in the two groups).

## Results

**Figure 1:**
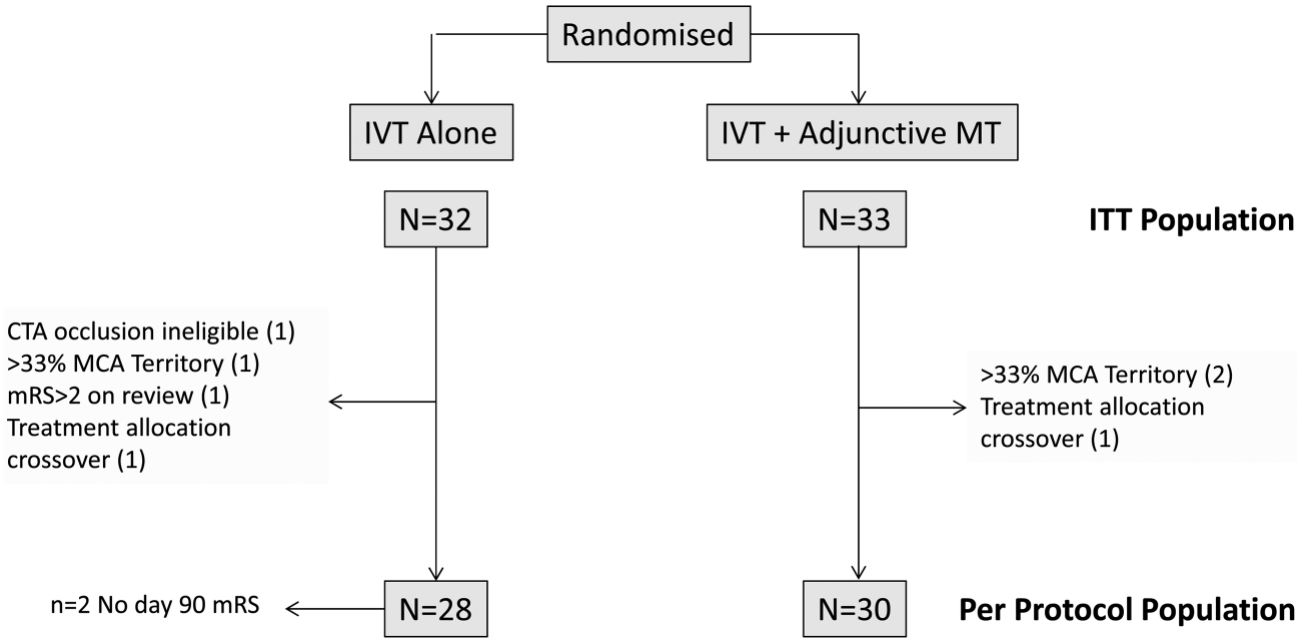
CONSORT flow chart showing disposition of trial participants. CTA, CT angiography; ITT, intention to treat; IVT, intravenous thrombolysis; MCA, middle cerebral artery; mRS, modified Rankin Scale; MT, mechanical thrombectomy.

Between April 2013 and April 2015, 65 patients were recruited at 10 centres in the UK. Trial recruitment was suspended in April 2015 following presentation of other relevant thrombectomy trial results and ended in June 2015. Seven patients were excluded from a per-protocol analysis based on major protocol deviations (figure 1). IVT alone was allocated to 32 patients and IVT with additional MT in 33. Two patients were lost to follow-up at day 90, with no mRS data available, both in the IVT-only group. Major demographic and medical history factors are detailed in table 1.

**Table 1:** Demographics, medical history, stroke characteristics and treatment process times

	Intravenous rtPA (IVT)	IVT+MT
n	32	33
Age, years, mean±SD	64±16	67±17
>80 years, n (%)	3 (9%)	6 (18%)
Male, n (%)	16 (50%)	13 (39%)
Estimated prestroke mRS		
0	28 (88%)	27 (82%)
1	1 (3%)	5 (15%)
≥2	3 (9%)	1 (3%)
Smoker (current), n (%)	3 (9%)	4 (12%)
MI or IHD, n (%)	6 (19%)	4 (12%)
Previous stroke, n (%)	2 (6%)	3 (9%)
Diabetes, n (%)	6 (19%)	11 (33%)
Hypertension, n (%)	17 (53%)	17 (52%)
Atrial fibrillation, n (%)	8 (25%)	15 (46%)
Prestroke antithrombotic therapy, n		
Aspirin	3 (9%)	2 (6%)
Clopidogrel	0	1 (3%)
Warfarin	2 (6%)	1 (3%)
Direct oral anticoagulant	0	3 (9%)
Glucose, mmol/L (mean±SD)	7.3 (3.4)	8.0 (3.2)
Pretreatment systolic/diastolic BP mm Hg (mean±SD)	144/83 (25/18)	147/77 (23/15)
NIHSS median, range	14 (6–29)	18 (6–24)
ASPECTS median, range	9 (2–10)	9 (4–10)
0–4, n (%)	1 (3%)	1 (3%)
5–7, n (%)	9 (28%)	6 (18%)
8–10, n (%)	22 (69%)	26 (79%)
CTA occlusion site, n (%)		
ICA T/L±M1±M2	6 (19%)	4 (14%)
MCA M1±M2	21 (65%)	22 (76%)
MCA M2	5 (16%)	3 (10%)
Collateral score, n (%)		
Good	12 (40%)	18 (55%)
Moderate	12 (40%)	10 (30%)
Poor	6 (20%)	5 (15%)
Extracranial ICA occlusion present, n (%)	1 (3%)	1 (3%)
Clot burden score, median (IQR)	6 (4, 7)	7 (4, 8)
Process times, min, median (IQR)		
Symptom onset to IVT start	120 (62, 238)	120 (61, 242)
Symptom onset to randomisation	150 (88, 268)	150 (78, 271)
IVT start to groin puncture		82 (28, 140)
Randomisation to groin puncture		58 (12, 87)
Groin puncture to device removal		49 (15, 137)
Total time, onset to procedure end		251 (181, 390)
Poststroke antithrombotic therapy, n		
Aspirin	17 (53%)	20 (61%)
Clopidogrel	10 (31%)	11 (33%)
Warfarin	1 (3%)	2 (6%)
Direct oral anticoagulant	2 (6%)	3 (9%)

ASPECTS, Alberta Stroke Programme Early CT Score; BP, blood pressure; CTA, CT angiography; ICA, internal carotid artery; IHD, ischaemic heart disease; IVT, intravenous thrombolysis; MCA, middle cerebral artery; MI, myocardial infarction; mRS, modified Rankin Scale; MT, mechanical thrombectomy; NIHSS, National Institutes of Health Stroke Scale; rtPA, recombinant tissue plasminogen activator.

Those randomised to receive MT were older, more often female, had more severe strokes, higher prevalence of some vascular risk factors (diabetes, atrial fibrillation) and a higher proportion had prestroke impairment on estimated mRS; a higher proportion had good collateral score and favourable ASPECT score (table 1).

### Procedural outcomes

Procedural timelines (table 1) were within protocol-recommended parameters. IVT was started a median of 120 min (IQR 93–150 min) after onset of symptoms. Among those allocated MT, interventional times were short and consistent with protocol recommendations. Total time from symptom onset to end of MT procedure was median 251 min.

Stent-retriever devices were used first in 68% of procedures and aspiration devices in 32%. General anaesthesia was used in 10/32 (31%) of patients and sedation in 22/32 (69%). In 25/32 (81%) patients (one patient did not undergo MT as randomised), a single device was used. TICI 2b-3 reperfusion at the end of MT procedure was achieved in 26/30 assessable immediate postprocedure angiograms (87%).

CTA at ∼24 hours was completed in 51/65 (78%) of patients and showed reduced likelihood of vessel occlusion among those randomised to additional MT compared to those treated with IVT alone. The proportion with an IST-3 score >1 (at least partial filling of major branches of the target vessel) was 77% vs 38%, and the proportion with IST3 score of 4 (complete patency, normal appearance) was 69% vs 33% (OR 0.18, 95% CI 0.05 to 0.64, p=0.008 in proportional odds regression of IST3 scores).

### Primary outcome

**Figure 2:**
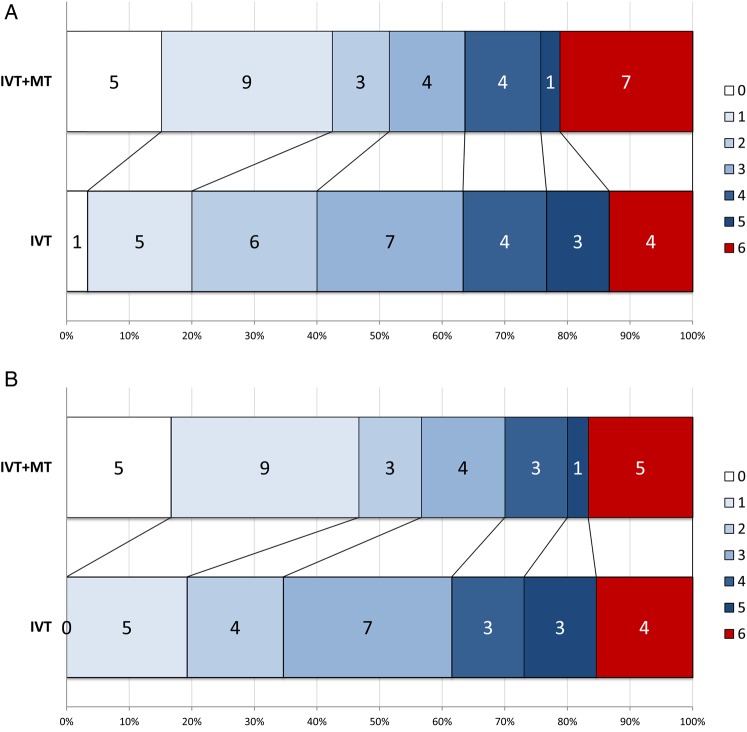
mRS distribution at day 90 in (A) ITT population and (B) per-protocol population. ITT, intention to treat; IVT, intravenous thrombolysis; mRS, modified Rankin Scale; MT, mechanical thrombectomy.

In the ITT population, the difference in the proportion achieving mRS 0–2 at day 90 (51% vs 40%, adjusted OR 2.12, 95% CI 0.65 to 6.94, p=0.204) was not significant. In the per-protocol population, however, there was a significant effect in favour of adjunctive MT after adjustment for minimisation variables, with an absolute difference in the proportion achieving mRS 0–2 at day 90 of 22% (57% vs 35%, OR 4.92, 95% CI 1.23 to 19.69, p=0.021) (table 2 and figure 2).

**Table 2:** Primary and secondary outcomes in ITT and per-protocol populations

	ITT		Per protocol	
mRS 0–2 at day 90	OR 2.12 (0.65 to 6.94)	p=0.204	OR 4.92 (1.23 to 19.69)	p=0.021
*Secondary outcomes*				
mRS 0–1 at day 90	OR 7.63 (1.56 to 37.22)	p=0.010	OR 14.6 (2.11 to 101.5)	p=0.005
mRS distribution	OR 2.59 (0.93 to 7.24)*	p=0.070	OR 4.47 (1.45 to 13.80)*	p=0.009
Death	OR 1.56 (0.29 to 8.40)	p=0.599	OR 0.69 (0.10 to 4.68)	p=0.697
Early major neurological improvement (NIHSS 0–1 or improved ≥8)	OR 1.83 (0.54 to 6.25)	p=0.321	OR 2.98 (0.76 to 11.65)	p=0.106
Days in usual residence, days 0–90	68 vs 78.5	p=0.782†	58 vs 79	p=0.411†
SICH (SITS-MOST)	0 vs 0	p=1.000‡	0 vs 0	p=1.000‡
PH1/2 ICH	1 vs 3	p=0.613‡	0 vs 3	p=0.238‡
IST-3 angiographic score=4 at 24 hours	OR 0.18 (0.05 to 0.64)	p=0.008	OR 0.17 (0.04 to 0.64)	p=0.009

*Adjusted for baseline (prestroke) mRS in addition to minimisation variables.

†p value from exact permutation test.

‡p value from exact Fisher test.

ITT, intention to treat; mRS, modified Rankin Scale; NIHSS, National Institutes of Health Stroke Scale; SICH, symptomatic intracerebral haemorrhage.

### Secondary efficacy outcomes

In the ITT population, there was a significantly greater likelihood of complete functional recovery (mRS 0–1) at day 90 with adjunctive MT compared to IVT alone after adjustment (OR 7.63, 95% CI 1.56 to 37.22, p=0.010). The difference in the distribution of mRS scores at day 90 (OR 2.59, 95% CI 0.93 to 7.24, p=0.070) did not reach significance (figure 2A and table 2).

In the per-protocol population, there was significantly greater likelihood of excellent outcome and better distribution of mRS scores at day 90 (figure 2B and table 2). The estimated number needed to treat for one person to have mRS ≤2 at day 90 was 6.91 in the adjusted analysis.

No significant difference in major early neurological recovery was seen in either ITT or PP populations, nor in the number of days spent in usual residence between stroke onset and day 90, although the direction of effects was consistently in favour of the additional MT group (table 2).

### Safety

In the ITT population, there were more deaths in the MT group (7 vs 4), but mortality did not differ significantly between IVT alone and IVT+MT groups (table 2). In the IVT group, one death was due to early brain swelling and three due to later complications (pneumonia or acute myocardial infarction (MI) 22–28 days after randomisation). In the IVT+MT group, four deaths were due to brain swelling and neurological deterioration (two of these in cases where recanalisation was not achieved and one in a patient excluded in the per-protocol analysis), one death from acute MI, one from aspiration pneumonia within the first week and one death due to pneumonia occurred later (27 days). There were no SICH events meeting SITS-MOST definition. Other ICH and adverse events are detailed in table 3. Three recurrent ischaemic stroke events in the MT group were not considered related to thrombus extraction in any case: two occurred 48–72 hours after the presenting event in patients with atrial fibrillation; the third occurred on the day of MT in a patient also in atrial fibrillation. The two patients with recurrent ischaemic stroke post-MT were on antiplatelet therapy only at the time of the event.

**Table 3:** Serious adverse events

	Intravenous rtPA (IVT)	IVT+MT
n	32	33
No. with any SAE	11 (34%)	15 (45%)
No. of SAEs reported	15	21
Probably or definitely related to study procedures	3	2
Fatal SAEs	4	7
No. fatal SAEs <7 days after onset	1	6
Fatal neurological events <7 days after onset	1	4
Intracerebral haemorrhage events		
Any ICH	3	3
SICH	0	0
ICH events on CT		
HI1 or HI2	12	13
PH1	1	1
PH2	0	2
Non-ICH SAEs	12	18
Anaemia	0	1
MI/acute coronary syndrome	2	1
Gingival bleeding	1	0
Pneumonia	4	5
Brain swelling	2	4
Recurrent ischaemic stroke	0	3
Neurological deterioration, not definitely ICH or swelling	0	1
Other	3 (CCF, UTI, psychiatric)	3 (pulmonary embolism, osteoarthritis, UTI)

CCF, congestive cardiac failure; HI1/HI2, haemorrhagic infarction types 1 or 2; ICH, intracerebral haemorrhage; IV, intravenous; MI, myocardial infarction; MT, mechanical thrombectomy; PH1/2, parenchymal haematoma types 1 or 2; rtPA, recombinant tissue plasminogen activator; SAE, serious adverse event; SICH, symptomatic intracerebral haemorrhage; UTI, urinary tract infection.

## Discussion

The PISTE trial was the only randomised controlled trial of MT in which a policy of proceeding as rapidly as possible to intervention on the basis of CTA confirmation of relevant LAO was pursued, in contrast to trials that either by protocol or in practice delayed endovascular treatment to assess the effects of IVT (MR CLEAN and REVASCAT)^10^ ^14^ or employed additional perfusion or intracranial collateral vessel imaging to select patients (ESCAPE, EXTEND-IA, SWIFT-Prime^11–13^). The findings are consistent with those reported from other clinical trials of MT. While the primary end point was not significant in the ITT population, a significantly greater proportion of patients allocated MT achieved the important secondary end point of excellent neurological recovery to mRS ≤1, and all major efficacy end points significantly favoured MT in the per-protocol population. The magnitude of estimated treatment effect was similar to those reported in other recent trials of MT (table 4).^25^

**Table 4:** Comparison of PISTE with published MT trials

	PISTE	MR CLEAN	ESCAPE	EXTEND IA	SWIFT Prime	REVASCAT
n	65	500	315	70	196	206
Key process times (MT arm), min, median						
Onset to IVT	120	85	110	127	110	117
Onset to randomisation	150	204	169	156	191	223
Onset to groin puncture	209	260	208	210	224	269
Onset to reperfusion	259	332	241	248	229*	355
TICI 2b-3	87%	59%	72%	86%	88%	66%
Absolute effect size						
mRS 0–1 at day 90	24%	6%	19%	23%	22%	12%
mRS 0–2 at day 90	14%	14%	25%	31%	24%	15.5%
mRS 5–6	2%	−7%	−14%	−23%	−17%	−6%
Mortality	9%	−1%	−9%	−11%	−4%	3%

*Estimated from interval times reported, but total times not reported.

IVT, intravenous thrombolysis; mRS, modified Rankin Scale; MT, mechanical thrombectomy; TICI, Thrombolysis in Cerebral Infarction.

Interventional procedures for acute ischaemic stroke have been undertaken increasingly in healthcare systems that reimburse these procedures since regulatory approval of endovascular devices from the mid-2000s.^26^ In contrast, few procedures have been undertaken in the UK, where interventional management of stroke has been uncommon, except in a small number of centres.

The efficacy of thrombectomy for large artery occlusive ischaemic stroke was first shown in a randomised trial in MR CLEAN^10^ and confirmed by results from four subsequently published trials (EXTEND-IA,^12^ ESCAPE,^11^ SWIFT-prime^13^ and REVASCAT^14^). Like PISTE, these four trials, and also two further endovascular trials that have been presented but not yet published (THRACE and THERAPY), were terminated prematurely after interim review of data by trial data monitoring committees in response to the MR CLEAN results. We continued recruitment to PISTE up until the presentation of THRACE in April 2015 since PISTE addressed a subtly different question compared to the other trials that had been published and MT was not an accepted standard of care in the UK until April 2016. Early discontinuation of the trial led to small sample size, which is likely to be the main factor in the lack of significant difference between groups for the primary end point, since process indicators do not suggest any significant difference in speed of intervention or effectiveness of the intervention (table 4). Our results are consistent with the benefit shown for MT in larger trials, including a significant increase in the proportion of patients achieving excellent recovery. As with previous studies, there were no safety issues, with respect to mortality, intracerebral haemorrhagic events or general adverse events.

MR CLEAN and REVASCAT delayed MT initiation in order to evaluate the effectiveness of intravenous rtPA—explicitly in the REVASCAT protocol, which stipulated a minimum 30 min delay in MT, and implicitly in MR CLEAN. Both trials were characterised by early initiation of IVT but then long delays to randomisation and intervention, and consequently later reperfusion than the other three published trials. Effect size estimates were somewhat lower. The three trials that did not delay MT, and recommended proceeding as fast as possible to intervention regardless of IVT, were more selective and all reported larger effect sizes and shorter reperfusion times, but interpretation is confounded by the use in each of these trials of additional advanced imaging selection using perfusion imaging^12^ ^13^ or ASPECTS+collateral imaging scoring.^11^ Of the two unpublished trials, THRACE predominantly used MRI for selection, and THERAPY used assessment of clot burden (requiring clot length >8 mm on CT). PISTE was the only trial to use simple imaging (CT and CTA) and a policy of proceeding as fast as possible to MT without additional imaging selection. Onset to reperfusion time was accordingly short, and comparable to the ‘complex imaging’ trials run in experienced MT centres.^11–13^

Previous trials have been based in well-organised regional or national networks (REVASCAT and MR CLEAN), and/or have selected sites that have significant experience of MT in addition to highly organised acute IVT services with rigorous centre credentialing.^11^ ^13^ It was important to establish whether similar effect sizes could be achieved in a healthcare setting where MT was not regarded as ‘standard of care’ and where the supporting networks were less developed. The findings of PISTE indicate that effect sizes similar to those achieved in the other MT trials are feasible within a less experienced organisational framework and without complex imaging, although the trial relied upon effective multidisciplinary teams within comprehensive stroke centres.

While it is important to regard findings based on small numbers with caution, the difference between the ITT and per-protocol analyses of PISTE suggests that adherence to strict patient selection criteria may be important in maximising the efficacy of MT. Those excluded from the per-protocol analysis included one crossover to MT in the IVT-only arm, five patients with inadequate assessment of preprocedure imaging (three more extensive established ischaemic change than permitted; one lack of vascular access to the target vessel due to extensive extracranial arterial occlusion; one ineligible occlusion site) and one patient with significant prestroke disability.

Although recent individual patient data and group-level meta-analyses, including the five published trials, have refined the effect size estimates and allowed some important subgroups to be clarified,^25^ there remain questions around generalisability, notably whether there are sufficient benefits in some groups of patients (eg, those with extensive early ischaemia, those ineligible for IVT), the minimum organisational and training requirements for safe and effective implementation, the role of advanced imaging selection and the cost-effectiveness of MT. Further clinical trials are required to investigate the limits of effectiveness for MT and to provide additional information on absolute effect sizes in different subgroups that will guide service implementation.

While we did not find significant differences in the primary outcome measure on ITT analysis, the secondary end point of excellent recovery (mRS 0–1) was significant in the ITT population, all mRS-based outcomes were significant in favour of MT in the per-protocol population and the effect size estimates were consistent with other trials. We therefore conclude that PISTE confirms the safety of a policy of adjunctive MT based on relatively simple imaging (CT+CTA) and supports striking benefit of MT in patients with acute large anterior circulation artery occlusive acute ischaemic stroke and the feasibility of such treatment within the UK healthcare system with well-organised services for delivery of IVT, but only limited prior experience of thrombus extraction for acute stroke.
